# Shear-induced acquired von Willebrand syndrome: an accomplice of bleeding events in adults on extracorporeal membrane oxygenation support

**DOI:** 10.3389/fcvm.2023.1159894

**Published:** 2023-07-06

**Authors:** Haiwang Wang, Duo Li, Yuansen Chen, Ziquan Liu, Yanqing Liu, Xiangyan Meng, Haojun Fan, Shike Hou

**Affiliations:** ^1^ Institute of Disaster and Emergency Medicine, Tianjin University, Tianjin, China; ^2^ Tianjin Key Laboratory of Disaster Medicine Technology, Tianjin University, Tianjin, China; ^3^ Wenzhou Safety (Emergency) Institute, Tianjin University, Wenzhou, China

**Keywords:** extracorporeal membrane oxygenation, bleeding, acquired von Willebrand syndrome, von Willebrand factor, centrifugal blood pump

## Abstract

Extracorporeal membrane oxygenation (ECMO) is an increasingly acceptable life-saving mechanical assistance system that provides cardiac and/or respiratory support for several reversible or treatable diseases. Despite important advances in technology and clinical management, bleeding remains a significant and common complication associated with increased morbidity and mortality. Some studies suggest that acquired von Willebrand syndrome (AVWS) is one of the etiologies of bleeding. It is caused by shear-induced deficiency of von Willebrand factor (VWF). VWF is an important glycoprotein for hemostasis that acts as a linker at sites of vascular injury for platelet adhesion and aggregation under high shear stress. AVWS can usually be diagnosed within 24 h after initiation of ECMO and is always reversible after explantation. Nonetheless, the main mechanism for the defect in the VWF multimers under ECMO support and the association between AVWS and bleeding complications remains unknown. In this review, we specifically discuss the loss of VWF caused by shear induction in the context of ECMO support as well as the current diagnostic and management strategies for AVWS.

## 1. A brief history and clinical applications of extracorporeal membrane oxygenation

**Figure 1:**
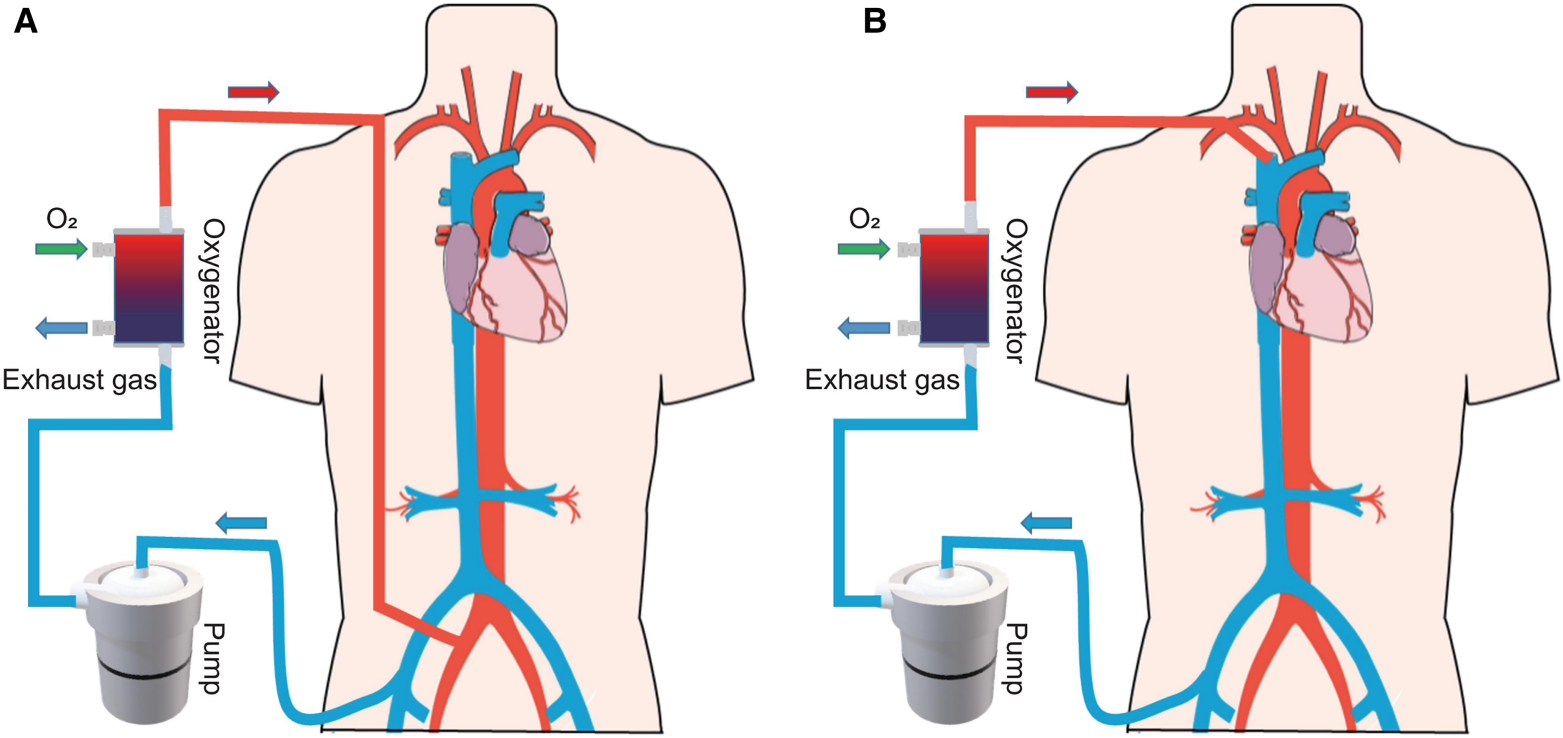
Schematic of ECMO system. (**A**) VA-ECMO and (**B**) VV-ECMO.

Extracorporeal membrane oxygenation (ECMO) is used to support critically ill patients with life-threatening cardiac and/or respiratory dysfunction when traditional treatment fails (1). The ECMO system consists of a pump to replace the function of the heart, while an oxygenator executes the work of the lungs. In clinical practice, the system includes two main configurations (Figure 1): venoarterial ECMO (VA-ECMO) and venovenous ECMO (VV-ECMO) (2). VA-ECMO, which drains the blood from a central vein and returns it to an artery, offers both respiratory and circulatory support (3). VV-ECMO, which drains the blood from a central vein and pumps it back to the central vein, supports gas exchange alone. In summary, the ECMO system offers temporary support, providing the heart and/or lung patient time to recover or undergo a transplant.

The first case report of successful ECMO in a patient with acute respiratory distress syndrome was published by Hill and colleagues in 1972 (4). With technological progress in pumps (5), oxygenators (6), and biocompatible coated surfaces (7), ECMO systems have been increasingly applied to support respiratory or cardiac function in clinical practice over the past few decades. However, ECMO has a high rate of complications, including bleeding, thrombosis, infection, and stroke (1). Particularly, bleeding complications are still a relevant problem despite improved equipment and survival rates.

## 2. Bleeding complications related to ECMO

### 2.1. Prevalence and trends of bleeding events

Despite significant improvements and technical developments in the ECMO systems, bleeding events, such as intracranial hemorrhage (ICH), pulmonary bleeding, gastrointestinal (GI) bleeding, and cannulation site bleeding, are common and associated with significant morbidity and mortality. According to the Extracorporeal Life Support Organization database on ECMO support from 2010 to 2017 (8, 9), the incidence rate of bleeding events was 62.1% and 37% in the VA-ECMO and VV-ECMO groups, respectively. Normally, bleeding occurring at the beginning of ECMO support is primarily caused by surgical and anticoagulation strategies such as cannulation and surgical site bleeding. This type of bleeding is called external bleeding in the clinic. Internal bleeding including ICH (10, 11) or GI bleeding (12–14) occurs later and usually has a more serious influence on the survival of patients on ECMO support (15). Mazzeffi et al. (16) reported that serious bleeding events occurred at a rate of 56.1% and red blood cell transfusion was also related to in-hospital mortality. In a prospective observational cohort study involving 514 patients on ECMO support, bleeding events occurred in 70.2% of the patients. These included internal bleeding in 16% of the patients, which was independently related to higher mortality (17).

### 2.2. Intracranial hemorrhage

The occurrence of ICH events was associated with higher mortality when compared with any other bleeding event in both VA-ECMO and VV-ECMO groups (18). According to the Extracorporeal Life Support Organization registry data, the incidence of ICH was 2.2% and 3.9% in the VA-ECMO and VV-ECMO groups, respectively (1). Patients experiencing ICH during ECMO support commonly have low survival rates and poor prognoses because of the complexity of diagnosis and treatment. Early diagnosis of ICH is critical in patients receiving ECMO support. However, some cases of ICH have been found by accident on brain imaging, which was performed along with thoracic/abdominal imaging or systematic screening. Lidegran et al. reported that 67% of the ICH cases were diagnosed within the first 4 days of ECMO support (19). Although some measures were taken according to the state of the patients after confirming ICH, the percentages of patients on VA-ECMO and VV-ECMO who experienced ICH and survived till discharge were only 11% and 26%, respectively, according to the Extracorporeal Life Support Organization registry (1). In conclusion, prompt recognition and optimal treatment are the most important factors for enhancing the outcomes in patients experiencing ICH while receiving ECMO support.

### 2.3. GI bleeding

GI bleeding is a significant problem that complicates ECMO support. It occurs in 10%–20% of the patients, increasing over time after using continuous-flow devices (20). Commonly, patients with clinical GI bleeding undergo colonoscopy, upper endoscopic assessment, and small bowel capsule endoscopy. Anticoagulation levels and antiplatelet regimen for patients can be adjusted according to the results of endoscopy to avoid ischemic stroke. However, some studies have demonstrated that persistent GI bleeding was observed in more than 52% of the cases despite adjustment of anticoagulation strategies, suggesting that anticoagulation might not be the only factor associated with bleeding complications in these patients (21). Therefore, new strategies in addition to anticoagulation should be developed to deal with GI bleeding.

## 3. Mechanisms of acquired von Willebrand syndrome

Acquired von Willebrand syndrome (AVWS) is a bleeding disorder caused by alterations in the structure or function of the von Willebrand factor (VWF). Due to the complexity of this illness and the continuous contact between the patient's blood and the extracorporeal circuit, the etiology of bleeding events is multifactorial and includes the heparin effect, coagulopathy, thrombocytopenia, platelet dysfunction, and AVWS. Some clinical and experimental data have demonstrated that AVWS in the bleeding events associated with left ventricular assist device (LVAD) is mainly caused by shear-induced reduction of high-molecular-weight VWF. Due to the similarity in centrifugal pump configuration between LVAD and ECMO, the pathogenesis of bleeding in ECMO is believed to contribute to the loss of high-molecular-weight VWF due to high shear stress (22).

### 3.1. Biology of VWF

VWF is a large, multimeric glycoprotein that executes two essential functions in hemostasis: mediating platelet tethering to the subendothelial matrix at sites of vascular injury and forming a complex with coagulation factor VIII in the blood plasma (23, 24). Patients who lack VWF exhibit a severe bleeding disorder because of defects in both platelet aggregation and blood clotting. Consequently, functional or quantitative defects in VWF are known as von Willebrand disease (VWD), which is a disorder affecting approximately 100 individuals per million (23). A congenital inheritance pattern is observed in VWD, while deficiency in VWF due to epigenetic factors is known as AVWS (25).

**Figure 2:**
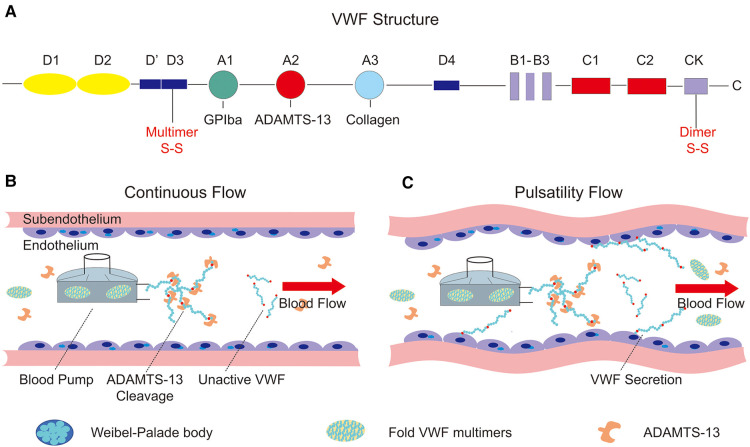
Structure of von Willebrand factor and the influence on the endothelium of continuous and pulsatility flow. (**A**) The structure of pre-pro-VWF and the conserved domains (A, B, C, D, CK), as well as the position of dimerization and multimerization; (**B**) continuous flow produced by a centrifugal pump. Supraphysiological shear stress generated by the driven device unfolds the VWF multimers, which cleavage by the ADAMTS-13 in blood flow; (**C**) the relationship between pulsatility and VWF secretion. The pulsatility of blood flow can stimulate the endothelial cells to secret VWF, which is stored in the Weibel–Palade body.

VWF is produced in endothelial cells and megakaryocytes and includes 2,813 amino acids as a single-chain pre-pro-polypeptide. The complete sequence was reported in 1986 and included four types of repeated motifs in the order D1-D2-D0-D3-A1-A2-A3-D4-B1-B2-B3-C1-C2-CK (Figure 2A). The A1 and A3 domains of VWF contain binding sites for platelets and subendothelial collagen, respectively (23). The A2 domain contains a Tyr^1605^–Met^1606^ peptide linkage that can be cleaved by a disintegrin-like metalloprotease with thrombospondin type 1 repeat 13 (ADAMTS-13) (26) which regulates the length of VWF multimers in blood. Domains B1–B3 and domains C1–C2 play key roles in the dimerization or multimerization processes by forming a dimeric bouquet (27, 28). The disulfide bond is located in the cysteine knot domain, which maintains the dimeric structure by connecting two monomers. Two dimers then multimerize through disulfide bonds in the D’D3 domain.

ADAMTS-13 is the main size regulator of VWF in blood flow and consists of 1,427 amino acid residues, comprising metalloprotease domain (M), disintegrin-like domain (D), type-1 thrombospondin domain (T), cysteine-rich domain (C), spacer domain (S), and CUB domain (29). Similar to VWF conformation, ADAMTS-13 presents a closed state in the absence of VWF binding under low shear stress, and it will cleave VWF when sufficient shear rates impose forces on VWF that expose its scissile bond (30, 31). Under normal conditions, circulating VWF multimers exist mainly in a collapsed state. Under conditions of higher shear stress than physiological level, the attractive forces between monomers are overcome by drag, and VWF unfolds abruptly and folds again, which allows for ADAMTS-13 cleavage in blood flow. In addition, newly released VWF multimers anchored on the surface of endothelial cells can be quickly cleaved by ADAMTS-13 in the blood, forming smaller multimers with a wide range of size distributions (32). After being released into the blood, these smaller multimers rapidly adopt a collapsed conformation, opposing the proteolysis of ADAMTS-13 in a normal physiological state (31). Collapsed VWF exposes the A2 domain to ADAMTS-13 under high shear stress conditions such as aortic stenosis (33), implantation of an LVAD (34), and ECMO support (35). Decreased VWF multimers and a reduction in the ratio between VWF activity and VWF antigens are associated with bleeding disorders.

Additionally, some studies have suggested that VWF is a powerful regulator of angiogenesis. Starke et al. demonstrated that short interfering RNA inhibition of VWF expression increased endothelial cell proliferation, migration, *in vitro* angiogenesis, and vascularization in VWF-deficient mice (36). *In vivo*, VWF regulates angiogenesis by mediating the storage of angiopoietin-2 and galectin-3. VWF can also bind to integrin αvβ3 and increase cell aggregation on the surface of vascular smooth muscle cells. Thus, excess cleavage of the VWF multimers by ADAMTS-13 interrupts blood vessel formation, resulting in a thin wall and fragile vascular network, which manifest as gastrointestinal bleeding in a clinical setting (37, 38).

### 3.2. Shear flow and the shape of VWF

After release from endothelial cells and regulation by ADAMTS-13, the concentration distribution of smaller VWF multimers reaches equilibrium within 2 h (23). In homeostasis, VWF multimers are irregularly coiled because of the self-association between VWF monomers (39). However, the steady state is easily altered because of changes in shear flow in the bloodstream. In shear flow, blood velocity is maximal at the center and reduces to zero near the vessel wall (40). In contrast, the gradient of the velocity or shear rate (denoted by inverse seconds, s^−1^) is maximal in the wall of a vessel and zero at the center. Generally, practical flow in vessels, especially in arteries, is composed of rotational and elongational components (41). The response to a collapsed VWF in the blood flow is a linear superposition of these two components. During normal stasis, the VWF multimers are coiled upon themselves, forming a collapsed structure with maximal diameters ranging from 60 to 200 nm (42). However, above a critical shear stress of 50 dyn/cm^2^, attractive forces between the domains of VWF monomers are overcome by hydrodynamic drag, and free VWF in the bloodstream elongates and tumbles (43, 44). Using hydrodynamic simulations, Alexander-Katz et al. (45) demonstrated that collapsed VWF multimers exhibit unfolding/refolding cycles at a critical shear rate.

### 3.3. The effect of shear stress on VWF

In circulation, a collapsed VWF multimer with its parts in the lamina elongates, since one part moves faster than the other under different (shearing deformation) velocities (43). However, the faster and slower parts of a VWF multimer change during tumbling. Therefore, hydrodynamic forces lead to the compaction of VWF multimers, a process increased by the attraction between the VWF domains (46, 47). VWF tumbles in the bloodstream with no adhesion and is subjected to periodic compaction and elongation. Due to the limited time available for the VWF multimer to unfold in each cycle, VWF does not interact with ADAMTS-13 or platelets in the bloodstream unless it experiences higher shear stress than normal (48).

In recent decades, there has been a significant change in the use of ECMO systems due to the shift from roller pumps to centrifugal pumps, which have a higher speed and continuous flow. When a VWF multimer passes the device and is exposed to high-speed impeller centrifugal pumps, it becomes susceptible to cleavage by ADAMTS-13 due to the unfolded VWF multimer exposing the A2 domain (49, 50). According to molecular dynamics simulations (51), the tensile force applied at the center of the protein is approximately 80 pN at a shear stress of 10 Pa and with 40 dimers. In addition, some clinical data have demonstrated that a tensile force above 10–15 pN can induce VWF cleavage by ADAMTS-13 or its adhesion with platelets (52). Therefore, the complications related to hemostasis and thrombosis could result from the high local shear stress in a centrifugal pump with a high impeller speed.

In other situations, shear-induced VWF adhesion to biomaterials or collagen could contribute to changes in the VWF structure. Therefore, VWF could be easier to unfold with this same force applied compared to free VWF in the blood, since immobilization of VWF further increases the force effect. Considering the size of a typical human platelet, this force is ∼76 τ when platelets adhere to VWF (52). Therefore, at the same shear stress of 10 Pa, the peak force applied to the VWF–platelet complex is as high as ∼760 pN. This force also triggers platelet activation and proteolysis induced by ADAMTS-13. In other words, VWF is subjected to different flows and is much more elongated in shear flow when compared with free VWF in blood.

Thus, there are two situations regarding the effects of mechanical stress and VWF multimers on blood flow under ECMO support. However, this discussion must be tempered, since the relationship between mechanical stress and AVWS is a hypothesis and not an established fact. In addition to the shear-induced unfolding of VWF, the role of mechanical stress in AVWS remains to be determined.

### 3.4. AVWS associated with ECMO

Some reports have demonstrated the development of AVWS in patients on ECMO support (35, 53–56). The main characteristics include the loss of large VWF multimers and reduced adhesion activities in the VWF–collagen and VWF–platelet interactions. This syndrome can be diagnosed immediately after ECMO implantation, and rapid recovery is possible after ECMO explantation. For example, Kalbhenn et al. (54) reported that all 59 patients treated with ECMO experienced severe AVWS after implantation and during therapy but recovered gradually within 3 h to 1 day after the explantation of ECMO support. Panholzer et al. (57) reported a 3-year cohort study and found a clinically relevant bleeding rate of 23% in patients with AVWS associated with blood group O, longer ECMO support time, and VV-ECMO cannulation. Recently, Hayakawa et al. (35) reported a patient with coronavirus disease who experienced a bleeding episode resulting in AVWS and disseminated intravascular coagulation under ECMO support. In addition, the AVWS after ECMO support is also presented in pediatric patients. Ruth et al. (58) conducted a retrospective research on AVWS in pediatric ECMO patients. They suggested that AVWS is an underdiagnosed disease in pediatric ECMO patients and the relevance between AVWS and bleeding disorder needed to be further researched. Therefore, it is important to prevent bleeding induced by AVWS during ECMO support to reduce the incidence of hemostatic complications.

There is a widespread belief that AVWS stems from high shear stress over the biophysical process due to turbulent flow and biomaterial surfaces within the ECMO support. This high shear stress from the extracorporeal circuit could unfold VWF and hasten the cleavage of ADAMTS-13, reducing the number of large VWF multimers and adhesive activity. This process has the same features as those observed in type 2A VWD (53). In addition, bleeding complications may be exacerbated by anticoagulation strategies during ECMO support. Therefore, improvements in novel pump technologies to reduce shear stress and novel anticoagulation strategies are important future research directions in the area of ECMO support.

## 4. Diagnosis of AVWS under ECMO support

Clinically, patients with AVWS usually present with defects in the VWF structure or function, with consequent mucocutaneous bleeding as well as excessive bleeding in response to surgery or trauma. These features are similar to those observed in type 2 VWD (3). Clinically, VWD is an inherited bleeding disorder and is divided into three types, i.e., types 1, 2, and 3 (59, 60). Approximately 70%–80% of the patients express type 1 VWD, which is characterized by a quantitative deficiency of VWF. Type 2 VWD, accounting for 20% of the patients, is characterized by the dysfunction of VWF caused by a normal or reduced VWF antigen concentration with the protein not fully functional. According to the nature of specific phenotypic, type 2 VWD is further divided into four types, i.e., types 2A, 2B, 2M, and 2N. Type 3 VWD, accounting for about 5% of the VWD patients, is rare but is the most severe bleeding disorder and is characterized by the absence of circulating VWF in plasma (61). In recent years, the classification of bleeding symptom severity in VWD has received considerable attention, and some new assessment tools have been developed (62, 63). However, there is no single diagnostic test for AVWS during ECMO support. Instead, a panel of laboratory tests is employed (Table 1). In these tests, anticipated abnormalities in the AVWS include a reduction in the ratio of VWF ristocetin factor to VWF antigen and loss of high-molecular-weight VWF multimers.

**Table 1:** Diagnostic assessment of acquired von Willebrand syndrome.

Assay	Description	Abnormality
VWF antigen (VWF:Ag)	VWF monomers without biological activity	Normal laboratory range varies Levels ≤50 IU/dl
VWF ristocetin cofactor assay (VWF:RCo)	The ability of VWF for binding to platelet glycoprotein Ib (GPIb) in the presence of ristocetin	Reduction with VWF:RCo being lower than VWF:Ag
VWF:collagen binding	The ability of VWF for binding to collagen; sensitive to the presence of high-molecular-weight multimers	VWF:collagen binding (VWF:CB)
VWF:RCo/VWF:Ag	Test the biological activity of available VWF for binding to platelets	≤0.65
VWF:CB/VWF:Ag	Test the biological activity of available VWF for binding to collagen	≤0.7
VWF multimers	Qualitative assessment of the size spectrum and banding pattern of VWF multimers	Intermediate and high-molecular-weight VWF multimers missing

The process of checking for unusual findings suggestive of AVWS during ECMO support requires further investigation. To date, AVWS appears to be a risk factor for bleeding, but this remains to be confirmed in future research.

## 5. Treatment of AVWS under ECMO support

### 5.1. Adjustment of blood speed

The treatment approach is based on the causes of AVWS, and the goal is to control and prevent bleeding. The approach employed to achieve these goals depends on the underlying mechanisms (64). Shear-induced loss of VWF multimers may be the main cause of AVWS occurring during ECMO support. Thus, it makes sense to adjust the speed of the pump or structure of the canal to decrease the turbulent flow. In some studies, device changes have been made to reduce shear stress by adjusting the design of the blood pump and restoring pulsatility by incorporating algorithms in the pump controller (65, 66).

### 5.2. Generating pulsatility through speed modulation of the blood pump

Despite the advantages of continuous-flow blood pumps, hemostatic complications occur more often than with older pulsatile-flow pumps, probably due to changes in both vascular endothelial physiology and hemodynamics. Therefore, some groups have reported effective results with added pulsatility in continuous-flow pumps depending on different control strategies. By modulating the pump speed of the continuous-flow LVAD, Soucy et al. (67) proposed a method to induce vascular pulsatility, thus improving cardiac function and perfusion of the end organ. In this study, asynchronous and synchronous speed modulations were studied and implemented in a bovine model of ischemic heart failure to test their availability. Hemodynamic and hematological assessments have verified the improvements in vascular pulsatility and end-organ perfusion achieved using the asynchronous speed adjustment method. In contrast, Vincent et al. (66) demonstrated the key function of vascular pulsatility in ECMO support by comparing continuous-flow and pulsatile-flow animal models and actual patients in the clinic. They shed light on the relationship between pulsatility and endothelial release of VWF, which might replenish VWF multimers and reduce bleeding complications. In addition, Wang et al. (68) and Fleck et al. (69), in their reports, demonstrated a novel pulsatile diagonal pump for pediatric extracorporeal life support system (ECLS) *in vitro* and in clinical practice. The results of them verified that the use of the Deltastream DP3 seems to be safe and effective for ECLS in children and there are no system-related complications in the cohort study. In conclusion, vascular pulsatility in the centrifugal pump can improve hemodynamic and end-organ perfusion as well as increase the secretion of VWF multimers from endothelial blood vessels (Figures 2B,C). The aforementioned studies demonstrated the importance of pulsatility in continuous-flow devices for mechanical circulation support.

### 5.3. Drug therapy

In addition to the aforementioned improvements in ECMO devices, some reports have discussed the use of medications for preventing AVWS in patients. The available treatments for efficacy and safety to address the bleeding disorder include desmopressins (70), VWF-containing concentrates (71), and intravenous immunoglobulins (71). Desmopressin is a synthetic analog of vasopressin and can be used to prevent and control bleeding disorder in some patients with AVWS. Generally, the dosage of desmopressin is 0.3 µg/kg over 30 min once or twice daily. In addition, VWF concentrate consists of plasma-derived VWF and recombinant VWF. In clinical practice (72), a 40 IU/kg dose of VWF concentrate can effectively improve VWF function in adult ECMO patients with AVWS. Meanwhile, this team (73) further demonstrated an *in vitro* test that high-dose (0.8 IU/ml) recombinant VWF may have an obvious effect on normal VWF function in ECMO patients and a minimal thrombotic risk compared to plasma-derived VWF concentration. So, we can choose the optimal type of VWF concentration according to the actual situation in clinical practice. Furthermore, the availability of intravenous immunoglobulins has been demonstrated in a clinical study in patients with AVWS, and the suggested dose is 1 g/kg daily for 2 days (74). In addition, some factor concentrates and antifibrinolytic drugs like the lysine analogs tranexamic acid (20–25 mg/kg per 8–12 h) and ε-aminocaproic acid (50–60 mg/kg per 4–6 h) can also be used as adjunct therapies together with desmopressin or VWF-containing concentrates for bleeding disorder of AVWS patients (75, 76).

In the presence of refractory GI bleeding during LVAD support, the indications for medications and checkpoints such as warfarin and international normalized ratio may be readjusted according to the complexity of patient-related factors and pump types (77). Octreotide and thalidomide have also been proven effective in inhibiting persistent GI bleeding, but research regarding their use in patients receiving ECMO support has been inadequate (78–81).

AVWS during ECMO support mainly occurs because of the super-physiological shear forces from the high speed of the impeller. Therefore, improvements aimed at decreasing the shear stress of the centrifugal pump and optimizing the canal structure to minimize any turbulent flow need to be investigated in the future.

## 6. Conclusion

In this review, we have summarized the history of ECMO and analyzed the AVWS-induced relevant hemostatic complications due to the defects in VWF multimer cleavage by ADAMTS-13. Although AVWS has been observed in nearly all patients receiving ECMO support, not all patients present with bleeding in the clinic (53). However, it is clear that AVWS in patients receiving ECMO support is associated with a higher risk of bleeding during treatment. Thus, to avoid hemostatic complications in patients on ECMO support, it is essential to consider the possibility of AVWS to perform diagnosis including VWF multimers test, VWF activity, and VWF antigen concentration (82) and achieve pulsatility in the continuous-flow blood pump. Continuous optimization of equipment and clinical management will help reduce the incidence of life-threatening bleeding in ECMO patients.
